# The PDE4 Inhibitor Tanimilast Restrains the Tissue-Damaging Properties of Human Neutrophils

**DOI:** 10.3390/ijms23094982

**Published:** 2022-04-29

**Authors:** Tiziana Schioppa, Hoang Oanh Nguyen, Valentina Salvi, Norma Maugeri, Fabrizio Facchinetti, Gino Villetti, Maurizio Civelli, Carolina Gaudenzi, Mauro Passari, Francesca Sozio, Ilaria Barbazza, Nicola Tamassia, Marco A. Cassatella, Annalisa Del Prete, Daniela Bosisio, Laura Tiberio

**Affiliations:** 1 Department of Molecular and Translational Medicine, University of Brescia, 25123 Brescia, Italy; tiziana.schioppa@unibs.it (T.S.); h.nguyen002@unibs.it (H.O.N.); valentina.salvi@unibs.it (V.S.); c.gaudenzi@unibs.it (C.G.); m.passari@unibs.it (M.P.); francesca.sozio@unibs.it (F.S.); ilaria.barbazza1@gmail.com (I.B.); annalisa.delprete@unibs.it (A.D.P.); 2 Autoimmunity and Vascular Inflammation Unit, IRCCS San Raffaele Scientific Institute, 20132 Milano, Italy; maugeri.norma@hsr.it; 3 Department of Experimental Pharmacology and Translational Science, Corporate Pre-Clinical R&D, Chiesi Farmaceutici S.p.A., 43122 Parma, Italy; f.facchinetti@chiesi.com (F.F.); g.villetti@chiesi.com (G.V.); m.civelli@chiesi.com (M.C.); 4 Department of Medicine, Section of General Pathology, University of Verona, 37124 Verona, Italy; nicola.tamassia@univr.it (N.T.); marco.cassatella@univr.it (M.A.C.)

**Keywords:** CHF6001, neutrophil extracellular traps (NETs), tumor necrosis factor-alpha (TNF-α), CXC motif chemokine ligand 8 (CXCL8), spontaneous apoptosis, human umbilical vein endothelial cells (HUVECs), elastase, myeloperoxidase (MPO), matrix metalloproteinase (MMP), budesonide

## Abstract

Neutrophils, the most abundant subset of leukocytes in the blood, play a pivotal role in host response against invading pathogens. However, in respiratory diseases, excessive infiltration and activation of neutrophils can lead to tissue damage. Tanimilast-international non-proprietary name of CHF6001—is a novel inhaled phosphodiesterase 4 (PDE4) inhibitor in advanced clinical development for the treatment of chronic obstructive pulmonary disease (COPD), a chronic inflammatory lung disease where neutrophilic inflammation plays a key pathological role. Human neutrophils from healthy donors were exposed to pro-inflammatory stimuli in the presence or absence of tanimilast and budesonide—a typical inhaled corticosteroid drug-to investigate the modulation of effector functions including adherence to endothelial cells, granule protein exocytosis, release of extracellular DNA traps, cytokine secretion, and cell survival. Tanimilast significantly decreased neutrophil-endothelium adhesion, degranulation, extracellular DNA traps casting, and cytokine secretion. In contrast, it promoted neutrophil survival by decreasing both spontaneous apoptosis and cell death in the presence of pro-survival factors. The present work suggests that tanimilast can alleviate the severe tissue damage caused by massive recruitment and activation of neutrophils in inflammatory diseases such as COPD.

## 1. Introduction

Neutrophils are the most abundant circulating leukocytes and the first responders of the host-protective innate immunity. The main mechanisms used by neutrophils to fight pathogens are phagocytosis, respiratory burst, degranulation, and release of extracellular DNA traps (NETs). NETs are web-like scaffolds of extracellular DNA containing histones and neutrophil granular proteins, such as myeloperoxidase and neutrophil elastase, that help neutrophils to immobilize and catch invading microbes, facilitating pathogen elimination [1]. During chronic neutrophilic diseases, excessive release of toxic proteases and reactive oxygen species correlates with dysregulated neutrophil recruitment, activation, and defective apoptosis. Recent evidence also showed a relationship between aberrant NET formation and tissue damage [2]. Thus, neutrophils can also play a role in perpetuating inflammation and tissue injury in chronic respiratory diseases such as chronic obstructive pulmonary disease (COPD) and asthma [2].

Phosphodiesterases (PDEs) are the major determinants of the intracellular cyclic nucleotide concentrations, and control the signals of these second messengers in a compartmentalized manner [3]. Among the different members of the wide family of PDEs, the type 4 isoforms (PDE4) are expressed by inflammatory cells such as dendritic cells, monocytes, neutrophils, and macrophages, where they play a major role by specifically inducing cAMP hydrolysis [4]. PDE4 inhibition decreases the release of inflammatory mediators and the expression of pro-inflammatory surface receptors by blocking the breakdown of cellular cAMP levels, thus restraining inflammation. As assessed by in vitro and in vivo studies, PDE4 inhibition effectively reduced the activation and recruitment of different immune cells including neutrophils [4]. Currently, the oral PDE4 inhibitor Roflumilast has been approved for use in a subset of patients with severe COPD and history of exacerbation episodes [5] on top of standard of care, which includes inhaled long-acting β2-agonist, muscarinic antagonist, and corticosteroids. Unfortunately, systemic administration of PDE4 inhibitors, including Roflumilast, is associated to several adverse effects that limit their use and doses in the clinical practice [6].

Tanimilast, originally known as CHF6001, is a novel PDE4 inhibitor designed for inhaled delivery to specifically target the respiratory tract with a good tolerability and safety profile, and a low incidence of PDE4 inhibitors class-related side effects [7]. In a Phase IIa study (ClinicalTrials.gov-NCT03004417), tanimilast decreased inflammatory markers in the blood and induced sputum of COPD patients. Previous works also indicated that tanimilast could decrease neutrophil recruitment both in vitro and in vivo in a mouse model of LPS-induced lung inflammation [8,9]. The present study was conceived to assess the effects of tanimilast on human neutrophil effector mechanisms.

## 2. Results and Discussion

### 2.1. Tanimilast Inhibits Human Neutrophil Adhesion to Resting Endothelial Cells

**Figure 1:**
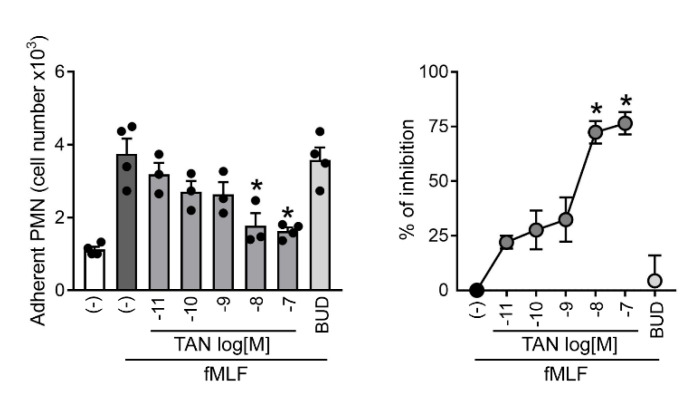
Tanimilast reduces neutrophil adherence to HUVEC monolayers. Human neutrophils, previously stained with CFSE, were incubated with the indicated concentrations of tanimilast (TAN), budesonide at 10^−7^ M (BUD), or vehicle (−), and then added to HUVEC monolayers in the absence or in the presence of fMLF 10^−6^ M. Neutrophil adhesion was assessed by counting neutrophils that remained associated to HUVEC after extensive washings, and were subsequently detached by trypsin-EDTA incubation. The left panel shows absolute cell numbers, while the right panel depicts the percentage of inhibition by tanimilast and budesonide of the neutrophil adherence found in fMLF-activated neutrophils. Dots represent the result of individual donors. * *p* < 0.05 One-way ANOVA followed by post-hoc test or one-sample *t*-test (*n* = 3–4).

The interaction of neutrophils with the endothelium represents a crucial step of the response to infection or tissue injury [10]. Thus, we set out to investigate the effects of tanimilast on neutrophil adhesion to endothelial cells. Upon triggering with fMLF (alias fMLP), a bacterial chemoattractant that also activates effector functions–including degranulation and production of reactive oxygen species–neutrophils acquired the ability to adhere to resting HUVEC. Accordingly, Figure 1 shows that in unstimulated conditions, only few neutrophils adhered to resting endothelium, possibly representing aspecifically-activated cells [11,12]. Upon fMLF challenge, the number of adherent neutrophils increased likely due to the increased expression of integrins which, in turn, bind cell adhesion molecules constitutively expressed by HUVEC [10]. Figure 1 shows also that tanimilast pre-treatment induced a dose-dependent decrease of the number of adhering neutrophils, which was statistically significant at concentrations of 10^−8^ and 10^−7^ M (left panel) with 72.4 ± 5.2 and 76.5 ± 5.1 percent of inhibition, respectively (right panel). Calculated IC50 was 1.97 nM, indicating a very high potency of tanimilast (Table 1). These results are in agreement with in vivo data obtained in a murine model of lung inflammation, showing that inhaled tanimilast can reduce the recruitment of neutrophils in bronchoalveolar lavage [8]. By contrast, the corticosteroid budesonide used at concentrations previously shown to inhibit several neutrophil functions [13,14] and active in inhibiting cytokine production (see below) could not counteract fMLF-induced adhesion to HUVEC (Figure 1). In this regard, previous reports demonstrated that another corticosteroid–dexamethasone–did not inhibit the binding of neutrophils to activated endothelial cells in vitro [15].

We are aware that this experimental setting cannot recapitulate neutrophil extravasation into inflamed lungs, especially due to technical limitations such as the use of umbilical vein- rather than pulmonary microvascular-endothelial cells, and the use of resting peripheral blood neutrophils. Indeed, the inhaled delivery of tanimilast suggests that, in vivo, it will likely predominantly act on extravasated cells. However, tanimilast was shown to reach the systemic circulation [16], likely through the pulmonary endothelium, where neutrophils could sense tanimilast before extravasation. In addition, HUVECs are merely used here as a physiological substrate to assess neutrophil adhesive properties as previously shown by Tonnesen et al. [17]. Thus, we think that our results may represent a proof of principle of the mechanisms underlying the reduction of neutrophil influx into inflamed pulmonary tissue in the presence of tanimilast.

### 2.2. Tanimilast Restrains the Release of Granule Proteins by Human Neutrophils

**Figure 2:**
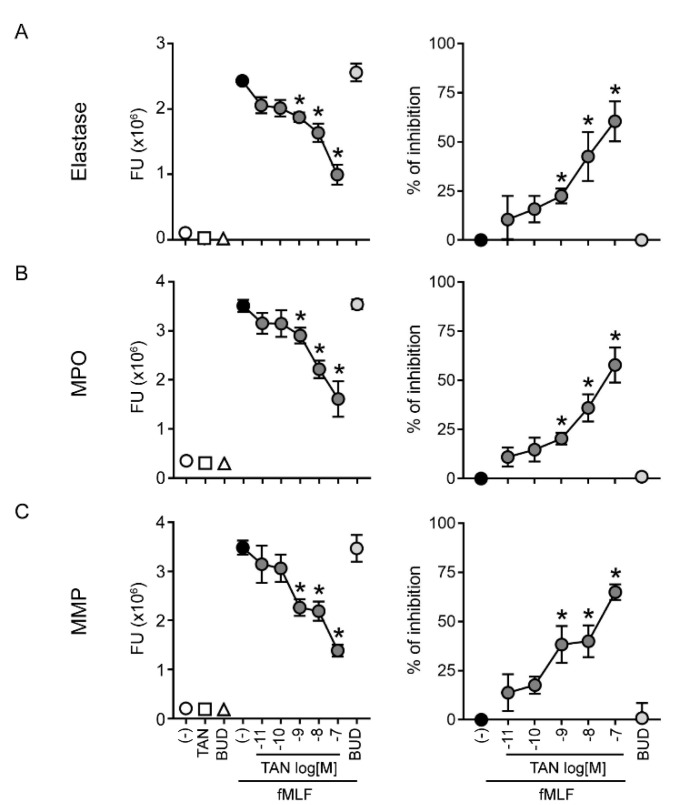
Tanimilast reduces neutrophil degranulation induced by fMLF. Human neutrophils were incubated with vehicle (−), tanimilast (TAN) at 10^−7^ M or as indicated, and budesonide (BUD) at 10^−7^ M. Degranulation was induced by fMLF treatment (2 × 10^−8^ M) in the presence of cytochalasin B. Granule protein release was assessed by measuring enzyme activities in cell-free supernatants, using elastase-specific (**A**), MPO-specific (**B**), or MMP-specific (**C**) fluorogenic substrates. Left panels show the fluorimetric readout (FU, Fluorimetic Unit) of each specific enzymatic activity in the cell supernatants, while right panels depict the calculated percentage of inhibition by tanimilast and budesonide. * *p* < 0.05 One-way ANOVA followed by post hoc test or One-sample *t*-test (*n* = 5).

Neutrophils exposed to fMLF also release granule proteins [11]. Granule protein release was assessed by measuring specific enzyme activities in cell-free supernatants. Tanimilast reduced in a dose-dependent manner the activity of elastase (Figure 2A), MPO (Figure 2B), and total MMPs (Figure 2C) released in the supernatants of fMLF-stimulated neutrophils. Calculated IC50s indicate that tanimilast acted in the nanomolar range (Table 1), with significant inhibition starting at the concentration of 10^−9^ M (Figure 2). Of note, at this concentration, tanimilast inhibited 40% of MMP activity, but only around 20% of elastase and MPO activity. Consistent with this, the inhibitory potency of tanimilast was significantly higher for MMPs as compared to elastase and MPO, the latter two showing superimposable IC50s (Table 1).

In the same experimental settings, budesonide did not inhibit granule protein release, in agreement with previous findings obtained with the corticosteroid drug dexamethasone [15].

Elastase and MPO are both contained in primary (azurophil) granules, while tertiary (gelatinase) granules contain abundant MMP-9 [11,18,19]. Granule mobilization is hierarchically regulated by the activation of specific signalling mechanisms, the triggering of which requires increasing stimulatory strength [20]. In particular, the content of gelatinase granules–which promotes neutrophil trafficking into the inflamed tissue via extracellular matrix degradation–is released early after activation, while histotoxic proteins contained in azurophil granules are released last, and degranulation requires the strongest stimulation [11,18]. This may explain the observed lower potency of tanimilast in inhibiting the release of elastase and MPO in respect to MMPs.

Aberrant neutrophil degranulation–with massive release of serine proteases, MMPs and MPO–contributes to the establishment of an increasingly proteolytic inflammatory milieu [21], which was also hypothesized to play a role in the pathogenesis of COPD [1,22,23]. Indeed, injection of elastase into animal lungs increased mucus secretion and altered its composition, leading to airways obstruction and development of emphysema [22]. Neutrophil elastase was also suggested to increase MMP activity by degrading endogenous MMP-inhibitors or directly activating MMP-9 [24,25], which in turn may further promote neutrophil recruitment via the production of bioactive by-products of extracellular matrix breakdown [26]. Thus, by reducing the release of granule proteins, tanimilast may prove beneficial in the treatment of diseases characterized by neutrophil-dominated inflammation. In addition, given the lack of budesonide effectiveness in reducing both adherence to endothelium and granule protein release, tanimilast may show a therapeutic advantage in conditions that are insensitive or resistant to glucocorticoids [27].

**Figure 3:**
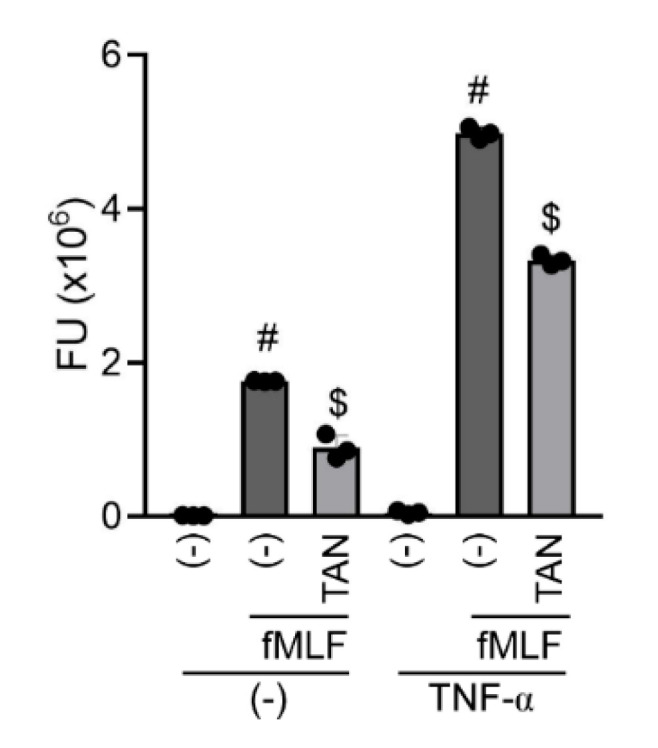
Tanimilast also counteracts elastase release by fMLF in the presence of TNF-*α*. Human neutrophils were incubated with vehicle (−) or tanimilast (TAN) at 10^−7^ M. When indicated, pretreatment with TNF-*α* (20 ng/mL) was also performed. Degranulation was induced by fMLF treatment (2 × 10^−8^ M) in the presence of cytochalasin B. Elastase release was assessed as in Figure 2. Results are expressed as fluorimetric readout (FU, Fluorimetic Unit) of elastase enzymatic activity in the cell supernatants. Percent of inhibition by tanimilast was 56.5 ± 4.5 and 33.5 ± 1.0 in the absence and in the presence of TNF-*α* priming, respectively. # *p* < 0.05 One-way ANOVA followed by post hoc test vs. fMLF untreated cells; $ *p* < 0.05 One-way ANOVA followed by post hoc test vs. fMLF-treated cells. (*n* = 3).

Our results also suggest that PDE4 inhibitors, by acting on intracellular cyclic AMP concentrations, may be more effective in interfering with rapid, membrane-originated events such as integrin activation and granule protein exocytosis as compared to glucocorticoids, which reduce inflammation mainly acting at the transcriptional level [27]. Previous studies have demonstrated that fMLF-induced azurophil degranulation is increased by treatment with priming agents such as TNF-α [12]. Since high amounts of TNF-α can be present in the blood or lung of COPD patients [28], the ability of tanimilast to inhibit elastase release in TNF-α-primed, fMLF-stimulated neutrophils was also analysed. Figure 3 shows that TNF-α priming did not directly activate neutrophil degranulation, but did significantly increase the elastase release by fMLF. Tanimilast also significantly inhibits fMLF-induced neutrophil degranulation in the presence of TNF-α, even if to a lower extent as compared to the inhibitory effect on unprimed, fMLF-stimulated neutrophils. In agreement with our data, previous studies showed decreased, but still significant, efficacy of other PDE4 inhibitors in counteracting neutrophil degranulation in the presence of TNF-α [12]. Overall, tanimilast also maintains efficacy in conditions where neutrophils are exposed to elevated TNF-α, such as COPD [28].

### 2.3. Tanimilast Reduces NET Production by Human Neutrophils

**Figure 4:**
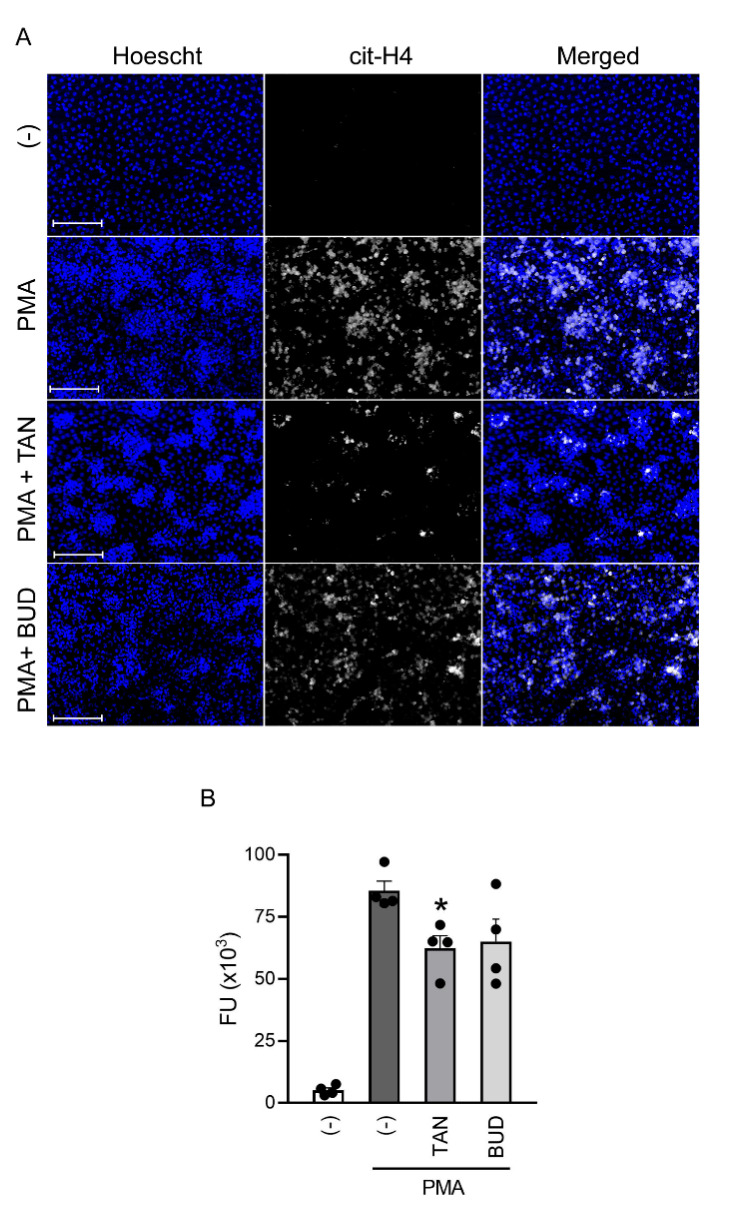
Tanimilast reduces NET production. Isolated neutrophils were left untreated (−) or exposed to PMA (100 ng/mL) in the presence of tanimilast (TAN) at 10^−7^ M, or budesonide (BUD) at 10^−7^ M. (**A**) NETs are identified by the association of citrullinated histon-4 (white) to DNA (blue). Scale bar: 100 μm. (**B**) Casted NETs were quantified in cell supernatants by analysing DNA-associated elastase activity after a limited DNase I digestion. Results are expressed as fluorimetric readout (FU, Fluorimetic Unit) of elastase activity in the cell supernatants. Dots represent the result of individual donors (*n* = 4). * *p* < 0.05% One-way ANOVA followed by post-hoc test.

To assess if tanimilast may affect NET formation, isolated human neutrophils were stimulated with PMA [29] in the presence or absence of the drug. Figure 4A shows that PMA stimulation was able to induce NETs, identified by intense citrullinated histone4 (cit-H4) positivity co-localizing with neutrophil DNA (Hoechst staining). No cit-H4 positivity could be detected in unstimulated cells. In the presence of tanimilast, NETs appeared less abundant, smaller, and more disperse. Similar results were observed in budesonide-treated cells. NET release was quantified in cell culture supernatants by measuring the enzymatic activity of DNA-associated elastase, as shown in Figure 4B. This quantification confirmed that tanimilast significantly reduced the amounts of NETs in the supernatants, while budesonide showed a variable effect among the different donors leading to a trend toward a reduction without reaching statistical significance. In the ongoing clinical trials in COPD patients, tanimilast has been proposed in association with cortocosteroid treatment [ClinicalTrials.gov-NCT03004417]. To analyse if tanimilast and budesonide can affect NET formation in a synergic manner, isolated neutrophils were exposed to PMA in the presence of both drugs (at 10^−7^ M). However, the combined treatment did not potentiate the effects of tanimilast alone (Figure S1).

NET formation was originally described as an anti-microbial effector mechanism of neutrophils [30] and, as such, represents a critical innate component of host defence against microbial invader. However, it is now clear that NET formation also plays major immunopathogenic roles [31]. Increased NET formation has been detected in the airways of COPD patients and in induced sputum of asthmatic patients, where it is associated with more severe disease outcomes and impairment of lung functions [32,33]. The observed reduction of NET casting by tanimilast may thus support its usage to reduce “lung NETopathy”, a term indicating NET-dependent tissue damage, that appears particularly relevant in the respiratory tract [2].

### 2.4. Tanimilast Inhibits the Secretion of Pro-Inflammatory Mediators by Human Neutrophils

**Figure 5:**
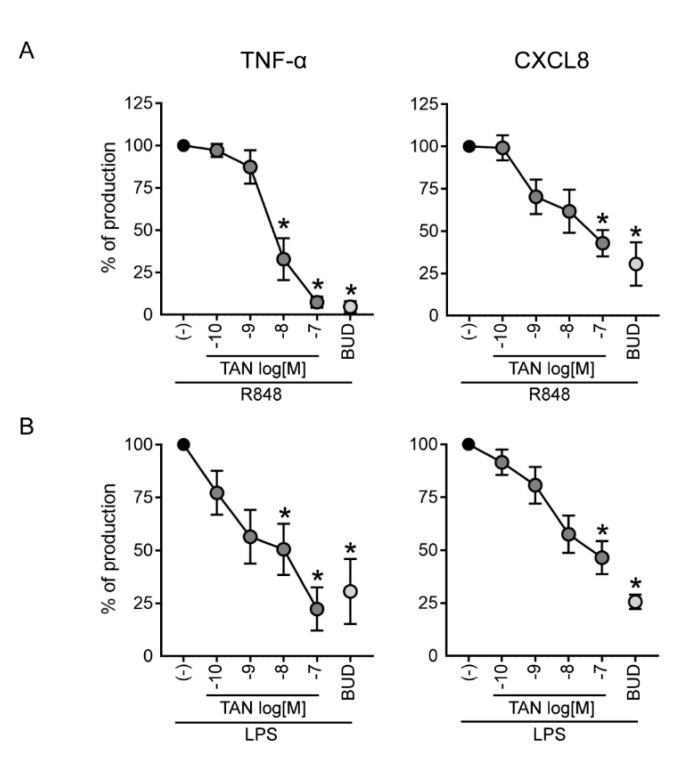
Regulation of pro-inflammatory cytokine secretion by tanimilast. Purified neutrophils were incubated with the indicated concentrations of tanimilast (TAN), budesonide at 10^−7^ M (BUD), and stimulated for 20 h with R848 (5 µM) (**A**) or LPS (100 ng/mL) (**B**). Graphs depict the calculated inhibition of the secretion of TNF-α (left panels) and of CXCL8 (right panels). The concentrations of secreted TNF-α were 73.7 ± 13.26 pg/mL after R484, and 24.6 ± 7.8 pg/mL after LPS; the concentrations of secreted CXCL8 were 3739 ± 702 pg/mL after R848, and 3186 ± 468 pg/mL after LPS. Results are expressed as the mean ± SEM from four independent donors. * *p* < 0.05 One Sample *t*-test.

Human neutrophils contribute to acute inflammatory diseases through the production and secretion of pro-inflammatory cytokines [34]. To evaluate if tanimilast can regulate this function, neutrophils were treated with R848–a potent TRL7/8 activator (Figure 5A)–and with LPS, the prototypic TLR4 ligand (Figure 5B); the release of TNF-α (left panels) and of CXCL8 (right panels) was evaluated in cell-free supernatants by ELISA [34]. Tanimilast dose-dependently reduced the secretion of both cytokines, but with a higher potency in the inhibition of TNF-α as assessed by the lower IC50 (Table 1). Consistent with previous literature [35], budesonide also inhibited the release of both cytokines, with an efficacy comparable to that of tanimilast. Similarly to what observed for NET formation, the combined treatment of tanimilast and budesonide (both at 10^−7^ M) did not lead to a further potentiation of the inhibitory effect obtained by the treatment with each drug alone (Figure S2).

These experiments were performed using highly purified neutrophils because of their low cytokine production on a per-cell base. In fact, even minor contamination by high cytokine-producing cells such as monocytes or dendritic cells, which can occur in standard neutrophil preparations, may give rise to false-positive results [34]. Indeed, cytokine production by LPS-stimulated monocytes and dendritic cells is also inhibited by tanimilast [36]. In inflamed tissues, however, the massive neutrophil infiltration compensates the limited cytokine producing capability of individual cells, thus rendering neutrophils key regulators of the inflammatory milieu. For example, activated neutrophils abundantly produce CXCL8, which self-perpetuates neutrophil recruitment [21]. Of note, CXCL8 is considered one of the most relevant chemokine in COPD, the levels of which are increased in sputum and directly correlate with the number of neutrophils in the lungs [37]. Thus, inhibiting the release of proinflammatory cytokines directly produced by neutrophils–in addition to those released by other innate cells–will positively affect the control of tissue inflammation. The super-maximal single concentration of budesonide (100 nM) utilized in this study, which almost completely ablates cytokines release, likely masks the potential additive effect of the combined treatment with tanimilast. Indeed, combined low-dose corticosteroid and low-dose tanimilast were previously shown to have a similar efficacy as high-dose corticosteroid [38]. It should also be considered that most of the severe COPD patients show poor response to the anti-inflammatory benefits of corticosteroids, a condition that cannot be mimicked in this in vitro experimental setting.

### 2.5. Tanimilast Promotes the Survival of Human Neutrophils

**Figure 6:**
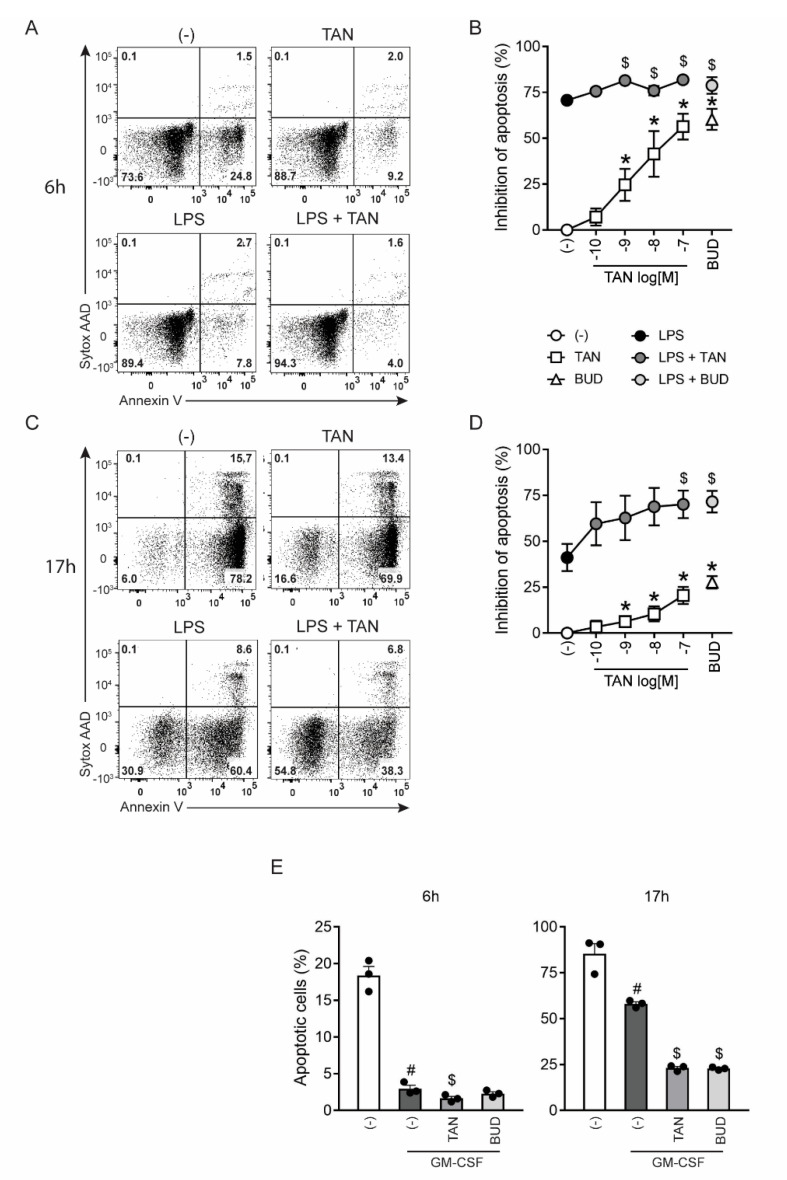
Tanimilast protects neutrophils from apoptosis. (**A**–**D**) Isolated neutrophils were exposed to the indicated concentrations of tanimilast and budesonide (10^−7^ M) in the absence or presence of LPS (500 ng/mL) (**A**–**D**). (**E**) Neutrophils were exposed to tanimilast or budesonide (both at 10^−7^ M) in the presence or absence of GM-CSF (20 ng/mL). Neutrophil apoptosis was assessed by cytofluorimetric analysis of Annexin V positivity in 7-AAD negative cells at 6 (**A**,**B**,**E** left panel) and 17 h (**C**,**D**,**E** right panel). (**A**,**C**) Dot plot from the analysis of one representative donor out of the four. (**B**,**D**) inhibition of apoptosis (%) by tanimilast (TAN) and budesonide (BUD), in unstimulated (open symbols) and LPS-stimulated neutrophils (closed symbols). Results are expressed as the mean ± SEM from the four independent donors. (**E**) Graphs depict the number of Annexin V positive in 7-AAD negative cells. Results are expressed as the mean ± SEM from three independent donors. Dots represent the result of individual donors (*n* = 3). * *p* < 0.05% One-Sample *t*-test; # *p* < 0.05% Student’s *t*-test; $ *p* < 0.05% One-way ANOVA followed by post-hoc test.

Neutrophils are short-lived cells that constitutively undergo apoptosis [39]. Thus, the effects of tanimilast were first assessed on spontaneous apoptosis. Budesonide was used as a positive control for apoptosis inhibition [14]. As expected, neutrophils rapidly underwent apoptosis reaching, at 6 h of culture, a mean percentage of apoptotic cells of 23.5 ± 4.3 (Figure 6A left upper panel and not shown). Additionally, in these experimental conditions, tanimilast inhibited spontaneous apoptosis (Figure 6A right upper panel) in a dose-dependent manner and up to 60% at 10^−7^ M (Figure 6B, white squares). Neutrophil apoptosis is delayed in the presence of pro-survival factors such as LPS or GM-CSF [40]. Accordingly, LPS stimulation for 6 h reduced the percentage of apoptotic neutrophils (mean percentage: 6.9 ± 1.5) (Figure 6A left lower panel and not shown). Tanimilast further increased neutrophil survival (Figure 6A lower right panel) in a dose-dependent manner (Figure 6B, grey circles).

The effect of tanimilast on neutrophil apoptosis was also assessed after 17 h of culture, when the percentage of apoptotic cells was 72.5 ± 1.71 in untreated samples and 50.2 ± 5.15 in the presence of the pro-survival factor LPS (Figure 6C left panels and not shown). Tanimilast rescued the cells from spontaneous apoptosis and potentiated LPS-induced cell survival (Figure 6C right panels) in a dose-dependent manner (Figure 6D). Similar protective effects were observed in the presence of the pro-survival growth factor GM-CSF (Figure 6E), suggesting that tanimilast modulates the lifespans of terminally differentiated granulocytes, independently of the nature of the pro-survival factor. Apoptosis inhibition by budesonide was comparable to the equimolar tanimilast concentration (Figure 6B,D, open triangle and light grey circle, and Figure 6E).

Conflicting results were reported concerning the effects of PDE4 inhibitors on neutrophil apoptosis. In vitro works, including ours, show that PDE4 inhibitors delay spontaneous apoptosis of cultured neutrophils [41,42]. In addition, we also show that tanimilast enhances the pro-survival effects promoted by microbial stimuli and cytokines. In contrast, in murine models of LPS-induced neutrophilic inflammation of the pleural cavity, PDE4 inhibition or cAMP increasing agents promoted the resolution of inflammation by increasing neutrophil apoptosis, as assessed by the analysis of cells recruited to the pleura [43,44]. As also suggested by these authors, both methodological and experimental factors could explain these contrasting data. Differently from in vitro experiments, the pharmacologic treatment has been performed on previously activated/migrated neutrophils, since it has been administered to animals at the peak of inflammation. In addition, in the in vivo milieu, both environmental (cytokines or other soluble factors) and cell-cell interactions may affect neutrophil fate or clearance, thus contributing to different and even opposite results [45].

## 3. Materials and Methods

### 3.1. Drugs and Treatments

Tanimilast and budesonide were kindly provided by Chiesi Farmaceutici S.p.A., (Parma, Italy). Budesonide was used as a typical inhaled corticosteroid drug utilized in COPD patients. Drugs were dissolved in sterile DMSO at 10 mM and further diluted in the experimental media. Final DMSO concentration was adjusted to be the same in all the samples (0.001%). Tanimilast concentrations were chosen as previously described [9,36].

### 3.2. Neutrophil Isolation

Buffy coats from healthy volunteers (through the courtesy of the Centro Trasfusionale, Spedali Civili di Brescia, Brescia, Italy) were used to isolate and purify neutrophils. Briefly, after Ficoll-Paque gradient centrifugation, granulocytes were isolated by dextran sedimentation and hypotonic lysis of erythrocytes yielding a neutrophil population with a purity higher than 90%, as assessed by flow cytometry using CD16/CD66b staining (Miltenyi Biotec, Bergisch Gladbach, Germany). For some experiments, to obtain higher neutrophil purity, the EasySep neutrophil enrichment kit (Stem cell Technologies) was used (>99%) [19].

### 3.3. Neutrophil Adhesion to HUVEC Monolayers

Adhesion experiments were performed as previously described [46] with minor modifications. Human umbilical vein endothelial cells (HUVECs), isolated from umbilical cords [47], were grown on culture plates coated with porcine gelatin in M199 medium supplemented with 20% FCS, endothelial cell growth supplement (100 µg/mL, Sigma-Aldrich), and porcine heparin (100 μg/mL, Sigma Aldrich, St. Louis, MO, USA). Cells at early (I–IV) passages were seeded at 5 × 10^5^ cells/cm^2^ in 96 well/plates and left undisturbed for 48 h. Isolated neutrophils were stained with CellTrace^TM^ CFSE (Thermo Fisher Scientific, Waltham, MA, USA) following manufacturer’s suggestions, resuspended in Hanks balanced salt solution (HBSS Ca^2+^ and Mg^2+^), and then incubated (10^5^ cells per 100 µL) with vehicle (DMSO), different concentrations of tanimilast (from 10^−11^ to 10^−7^ M), or budesonide (10^−7^ M) for 30 min. Neutrophils were then added to HUVEC in the presence or in the absence of 10^−6^ M N-formyl-methionyl-leucyl-phenylalanine (fMLF alias fMLP). After 30 min, non-adherent neutrophils were removed by several washes with HBSS. Adherent cells were detached by the incubation with trypsin-EDTA and CFSE-stained neutrophils were counted taking advantage by the volumetrical injection system of the MacsQuant 16 cytofluorimeter (Miltenyi Biotec, Bergisch Gladbach, Germany), which allows an absolute volumetric count.

### 3.4. Degranulation Assay

Neutrophil degranulation was induced as previously described [12], with minor modification. Briefly, isolated neutrophils were resuspended in HBSS Ca^2+^ and Mg^2+^ containing 1% human serum albumin (HSA) and were incubated with different concentrations of the tested compounds or vehicle control in 96 well polypropylene plates. After 30 min, cytochalasin B (5 µg/mL) was added to each sample for a further 30 min, followed by fMLF stimulation (2 × 10^−8^ M). In some experiments, TNF-α (20 ng/mL) was also added alongside cytochalasin B before fMLF treatment. After 45 min, plates were centrifuged, and cell-free supernatant containing degranulated proteins was collected. Next, fMLF-induced degranulation was measured by determining elastase, myeloperoxidase (MPO), and matrix metalloproteinase (MMP) enzymatic activities in the supernatants using specific fluorogenic substrates, as previously described [48,49,50]. As the fluorogenic substrate used to determine MMP activity (OMNIMMP, Enzo Life Sciences, Farmingdale, NY, USA) is adequate for nearly all MMP enzymes, it allows the evaluation of the “total MMP” activity.

### 3.5. NET Release

NET release was visualized as described [51]. Isolated neutrophils (5 × 10^6^ cell/mL) were seeded on polylysine-coated glass slides and left adhering for 30 min at 37 °C. Then, PMN were treated with tanimilast (10^−7^ M) or budesonide (10^−7^ M) or vehicle (DMSO) (Sigma-Aldrich, Germany) for 30 min and challenged with PMA (100 ng/mL) for 1 h. Next, cells were fixed with 1% paraformaldehyde (Pierce, Thermo Fisher Scientific), and incubated with a rabbit anti-citrullinated–histon4 antibody (Ab81797, Abcam), followed by an Alexa-488- conjugated anti-rabbit antibody (Thermo Fisher Scientific). Hoechst 33,342 dye was used to counterstain cell DNA. NETs were visualized with the fluorescence microscopy Zeiss Observer.Z1 at a magnification of 200× and Apotome2 for optical sectioning. Images were acquired using AxioVision software. NET quantification was performed by analysing the DNA-associated elastase activity. To this purpose, neutrophils (2.5 × 10^6^ cells/mL) seeded in a 96 well/culture plate were pretreated with tanimilast (10^−7^ M), budesonide (10^−7^ M) or vehicle (DMSO) for 30 min and then exposed to PMA (100 ng/mL). After 4 h, wells were washed to remove soluble elastase and DNAse I (1 U/mL) was added in order to digest extracellular DNA and free NET-adsorbed elastase. Finally, 10 min later, DNase activity was blocked by EDTA, and cell supernatants were collected, centrifuged and elastase activity was assessed as described earlier.

### 3.6. Cytokine Production

Purified neutrophils (5 × 10^6^ cell/mL) [19], cultured in RPMI containing 10% FCS, were stimulated with R848 (5 µM) or LPS (100 ng/mL) for 20 h, in the presence of different concentrations (from 10^−10^ to 10^−7^ M) of tanimilast or the fixed concentration of 10^−7^ M of budesonide (Sigma-Aldrich, Germany). Vehicle (DMSO) (Sigma-Aldrich, Germany) was added in unstimulated or stimulated controls. The secretion of CXCL8 and TNF-α was investigated by ELISA on cell free culture supernatants [52] by using DuoSet^®^ ELISA kit (DuoSet^®^ ELISA Development System, R&D System).

### 3.7. Assessment of PMN Apoptosis

Neutrophils were seeded in a 96-well polypropylene plate at 10^6^/^mL^ in RPMI containing 1% HSA and treated with different concentrations (from 10^−10^ to 10^−7^ M) of tanimilast or a fixed concentration of budesonide (10^−7^ M). For spontaneous apoptosis detection, neutrophils were incubated for 6 or 17 h. In another set of experiments, treated cells were exposed to LPS (500 ng/mL) or GM-CSF (20 ng/mL) for 6 or 17 h. At the end of the incubation, cells were stained with fluorescent-Annexin V and 7-AAD kit (BioLegend) following the manufacturer’s instructions, acquired in a MacsQuant 16 cytofluorimeter (Miltenyi Biotec), and analysed using FlowJo software (BD Biosciences, Eysins, Switzerland).

### 3.8. Statistics

Comparisons among treatments were performed by One-way ANOVA followed by Dunnett’s post-hoc tests; normalized data have been compared by One-sample *t*-test. IC50 was calculated by nonlinear regression by using the least-square ordinary fit and -log(inhibitor) vs. response-equation. Comparisons between regressions were performed by the extra sum-of-square F test. *p* < 0.05 was considered statistically significant. GraphPad Prism program was used for calculations.

## 4. Conclusions

Inhibitors of the phosphodiesterase 4 (PDE4) enzyme–like the oral drug roflumilast–have shown a potential to reduce inflammation-mediated processes, and the frequency of exacerbations in certain groups of COPD patients with a chronic bronchitis phenotype [53]. To unravel the effects of the novel inhaled PDE4 inhibitor tanimilast on neutrophil effector functions with the view of its usage in COPD, neutrophils were challenged with bacterial or viral-mimetic agonists. Our results, in short, demonstrate that tanimilast modulates neutrophilic adhesion to endothelial monolayers, cytokine production, degranulation, apoptosis, and NET formation.

Of note, the reported inhibition of CXCL8, TNF-α, and MMP9 mirrors previous data obtained in induced sputum of COPD patients treated with tanimilast [16], underlying the translational significance of the present research, and providing evidence of a direct effect of the drug on neutrophil functions.

Additionally, unlike tanimilast, budesonide–an inhaled corticosteroid widely utilized in COPD–did not reduce endothelial adhesion and degranulation, suggesting that modulatory effects of corticosteroids and PDE4 inhibitors on neutrophils are not redundant. This study provides a mechanistic insight underlying the beneficial effects of tanimilast, as well as PDE4 inhibitors in general, in the treatment of lung pathologies–such as COPD–characterized by neutrophil infiltration, and supports a potential advantage in conditions that are insensitive or resistant to glucocorticoids.

## Figures and Tables

**Table 1:** Inhibitory potency of tanimilast on different neutrophil functions.

Function	Stimulus	IC50 (nM)
Adhesion to endothelium	fMLF	1.97
Degranulation:		
NE	fMLF	49.2 ^#^
MPO	fMLF	48.8 ^§^
MMP	fMLF	9.80 ^#,§^
Cytokine secretion:		
TNF-α	R848	53.3 °
	LPS	145 *
CXCL8	R848	8.36 °
	LPS	4.20 *
Spontaneous apoptosis inhibition (6 h)	NONE	39.5

^#,§,^*^,^° *p* < 0.05 Extra sum-of-square F test. The values with the same symbols are significantly different.

## Data Availability

The original contributions presented in the study are included in the article/supplementary material. Further inquiries can be directed to the corresponding author.

## Supplementary Materials

The following supporting information can be downloaded at: www.mdpi.com/article/10.3390/ijms23094982/s1.
