# Supplementary material for: The PDE4 Inhibitor Tanimilast Restrains the Tissue-Damaging Properties of Human Neutrophils
Source: Int J Mol Sci. 2022 Apr 29;23(9):4982. doi: 10.3390/ijms23094982 (PMC9104715; doi:10.3390/ijms23094982)
Supplement: Supplementary file 1 [file ijms-23-04982-s001.zip › ijms-1684813-supplementary.pdf]

The PDE4 Inhibitor Tanimilast Restrains the Tissue-Damaging Properties of Human Neutrophils

Supplementary

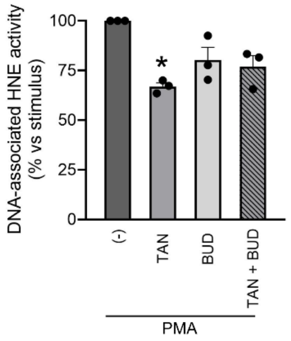

Figure S1. Cotreatment with budesonide does not potentiate tanimilast inhibition of NET production. Isolated neutrophils were left untreated (−) or exposed to PMA (100 ng/ml) in the presence of tanimilast (TAN) at 10^{-7}M or budesonide (BUD) at 10^{-7}M or both (TAN+BUD) at 10^{-7}M. Casted NETs were quantified in cell supernatants by analysing DNA-associated elastase (HNE) activity after a limited DNase I digestion. Results are expressed as fluorimetric readout (FU, Fluorimetic Unit) of elastase activity in the cell supernatants. Dots represent the result of individual donors (N=3). **p* < 0.05% One-sample t-test.

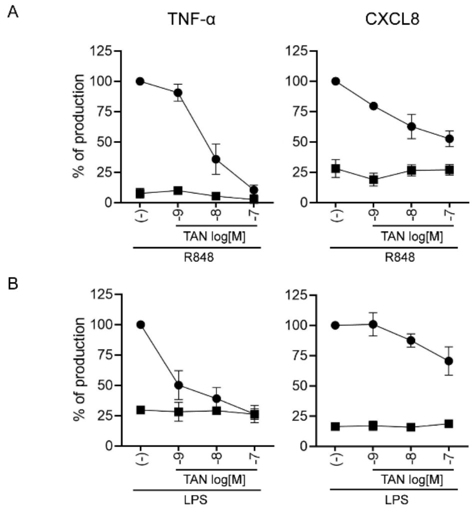

Figure S2. Cotreatment with tanimilast does not increase the already nearly-maximal inhibitory effect of budesonide against cytokine production. Purified neutrophils were incubated with vehicle (-) or tanimilast (TAN) at the indicated concentrations, in the absence (closed circles) or in the presence (closed squares) of budesonide at 10^{-7}M and stimulated for 20 hours with R848 (5 µM) (**A**) or LPS (100 ng/ml) (**B**). Graphs depict the calculated inhibition of the secretion of TNF-α (left panels) and of CXCL8 (right panels). Results are expressed as the mean±SEM from three independent donors.
